# Patient Experiences and Perceptions Associated with the Use of Desiccated Thyroid Extract

**DOI:** 10.3390/medicina56040161

**Published:** 2020-04-03

**Authors:** Freddy J. K. Toloza, Nataly R. Espinoza Suarez, Omar El Kawkgi, Elizabeth H. Golembiewski, Oscar J. Ponce, Lixia Yao, Spyridoula Maraka, Naykky M. Singh Ospina, Juan P. Brito

**Affiliations:** 1 Knowledge and Evaluation Research Unit, Mayo Clinic, Rochester, MN 55902, USA; ftolozabonilla@uams.edu (F.J.K.T.); EspinozaSuarez.Nataly@mayo.edu (N.R.E.S.); ElKawkgi.Omar@mayo.edu (O.E.K.); Golembiewski.Elizabeth@mayo.edu (E.H.G.); ponceoscarj@gmail.com (O.J.P.); smaraka@uams.edu (S.M.); nsinghospina@ufl.edu (N.M.S.O.); 2 Division of Endocrinology and Metabolism, University of Arkansas for Medical Sciences, Little Rock, AR 72205, USA; 3 Division of Endocrinology, Diabetes, Metabolism and Nutrition, Mayo Clinic, Rochester, MN 55902, USA; 4 Division of Digital Health Sciences, Department of Health Sciences Research, Mayo Clinic, Rochester, MN 55902, USA; yao.lixia@mayo.edu; 5 Central Arkansas Veterans Healthcare System, Little Rock, AR 72205, USA; 6 Division of Endocrinology and Metabolism, University of Florida, Gainesville, FL 32611, USA; 7 Plummer 3-35, 200 First Street SW, Rochester, MN 55905, USA

**Keywords:** hypothyroidism, desiccated thyroid extract, levothyroxine, therapy

## Abstract

*Background and objectives:* It is unclear why many patients with hypothyroidism prefer the use of desiccated thyroid extract (DTE) as a thyroid hormone replacement formulation over levothyroxine (LT4) treatment, as recommended by clinical practice guidelines. We analyzed patient-reported information from patient online forums to better understand patient preferences for and attitudes toward the use of DTE to treat hypothyroidism. *Materials and Methods:* We conducted a mixed-methods study by evaluating the content of online posts from three popular hypothyroidism forums from patients currently taking DTE (*n* = 673). From these posts, we extracted descriptive information on patient demographics and clinical characteristics and qualitatively analyzed posts’ content to explore patient perceptions on DTE and other therapies further. *Results:* Nearly half (46%) of the patients reported that a clinician initially drove their interest in trying DTE. Patients described many reasons for switching from a previous therapeutic approach to DTE, including lack of improvement in hypothyroidism-related symptoms (58%) and the development of side effects (22%). The majority of patients described DTE as moderately to majorly effective overall (81%) and more effective than the previous therapy (77%). The most frequently described benefits associated with DTE use were an improvement in symptoms (56%) and a change in overall well-being (34%). One-fifth of patients described side effects related to the use of DTE. Qualitative analysis of posts’ content supported these findings and raised additional issues around the need for individualizing therapy approaches for hypothyroidism (e.g., a sense of each patient has different needs), as well as difficulties obtaining DTE (e.g., issues with pharmacy availability). *Conclusions:* Lack of individualized treatment and a feeling of not been listened to were recurrent themes among DTE users. A subset of patients may prefer DTE to LT4 for many reasons, including perceived better effectiveness and improved overall well-being, despite the risks associated with DTE.

## 1. Introduction

Hypothyroidism affects 0.5–2% [1,2,3] of the population in the United States and leads to multiple symptoms, including fatigue, weight gain, dry skin, constipation, poor memory, depression, and overall decreased quality of life. Levothyroxine (LT4), a synthetic thyroid hormone, is the recommended treatment for patients with hypothyroidism [4,5,6]. LT4 effectively restores the levels of thyroid hormone and improves thyroid-related symptomatology. However, despite the strong evidence regarding the effectiveness of LT4 for the treatment of hypothyroidism, some patients prefer other thyroid hormone replacement formulations. Although it is not approved by the Food and Drug Administration (FDA), one of the most commonly used non-LT4 formulations for the treatment of hypothyroidism is desiccated thyroid extract (DTE) (e.g., nature thyroid, thyroid USP, and Armour thyroid), preparations made from desiccated pig thyroid glands. It is estimated that about 10–29% of patients with hypothyroidism use DTE as their primary thyroid hormone replacement medication in the US [7,8], despite concerns about the potential risk of thyrotoxicosis associated with DTE use [9,10]. It is unclear why many patients prefer the use of DTE over LT4 as a thyroid hormone replacement formulation.

A previous survey conducted among patients with hypothyroidism identified an overall high dissatisfaction rate (10-points Likert scale) with therapy for hypothyroidism (5 [Interquartile range (IQR) = 3–8]); however, when stratified by treatment modality, patients taking DTE showed less treatment dissatisfaction (7 [IQR = 4–8]) compared to the individuals treated with LT4 (5 [IQR = 3–7]) [8]. Although this study showed that higher treatment satisfaction with DTE therapy does exist among patients with hypothyroidism, there remains a limited understanding of factors that may be driving patients to choose DTE over LT4 treatment among patients with hypothyroidism, despite there being no evidence to support using DTE in preference to LT4 monotherapy in the treatment of hypothyroidism [4].

Patient-shared experiences in online health communities (i.e., social media and patient forums), as an easily accessible and efficient data source, have been widely used to assess treatment effects, adherence, and perceived quality of care from the end-user perspective [11,12,13,14,15,16,17,18,19]. Through these online health communities, patients have the opportunity to share their experiences, perceptions, feelings, and options with their peers. Therefore, in this study, we analyzed patient-reported information from patient online forums to better understand patient preferences for and attitudes toward the use of DTE to treat hypothyroidism.

## 2. Materials and Methods

The study was considered exempt by the Institutional Review Board at the Mayo Clinic, as no identifiable participant information was collected. Data retrieval of anonymous posts in publicly accessible patient discussion forums from patients treated with DTE was employed. We searched the ten most popular patient forums, based on the number of users (*WebMD, Topix, Health questions, PatientsLikeMe, Drugs.com, Endocrine Web, Everyday Health, Talk Health Partnership, Spark People,* and *Patients.info*), using the following key terms: desiccated thyroid extract, desiccated thyroid treatment, Armour Thyroid or Nature Thyroid (commercial names of DTE), thyroid extract, and hypothyroidism. A total of 1235 unique posts were retrieved from those websites, between each forum’s inception to March 2018. After the initial screening, the total 673 posts from three of these forums (*WebMD, PatientsLikeMe,* and *Drugs.com*) were selected for analysis, based on the completeness of the available information (hypothyroidism etiology, age, sex, DTE dose, and quantitative report of perceived effectiveness with DTE therapy) in these forums. *WebMD* (first post: October 2007) is an online publisher of news and information about human health and well-being, including drug information and user-based reviews and comments. *PatientsLikeMe* (first post: October 2004) is a network that connects patients with others who have the same disease or condition and track and share their own experiences. *Drugs.com* (first post: May 2008) is an online pharmaceutical encyclopedia that provides drug information and user reviews/comments for consumers and healthcare professionals. Posts’ content was downloaded to spreadsheets for analysis purposes, and three independent reviewers extracted data from each of these posts in duplicate.

### 2.1. Quantitative Analysis

In order to analyze relative frequencies of patient perceptions and attitudes, we extracted the following information from the retrieved posts: treatment indication (e.g., Hashimoto’s thyroiditis and post-surgical hypothyroidism); patient gender; patient age; DTE dosage; patient-perceived DTE treatment effectiveness; adverse effects from and benefits of DTE; DTE treatment duration; source of DTE initiation (clinician-initiated or patient-initiated); sources of information about DTE; sources of obtaining DTE; and characteristics, benefits, and side effects of any treatments used for hypothyroidism prior to DTE. Regarding patient-perceived effectiveness of DTE, the posts contained either a categorical (*PatientsLikeMe*: major, moderate, slight, or none) or numerical scale of DTE effectiveness (*WebMD*: 1 to 5 scale; Drugs.com: 1 to 10 scale). We codified the numerical scales as a categorical variable, as follows: *WebMD*: 5 = major, 4 = moderate, 2–3 = slight, 1 = none; Drugs.com: 9–10= major, 6–8 = moderate, 3–5 = slight, 1–2 = none. Summary statistics are presented as frequencies (percentages) for the categorical variables and as means and standard deviation (SD) for continuous variables.

### 2.2. Qualitative Analysis

Additionally, to complement quantitative findings, a random sample of 40% of posts including more than ten words was selected for qualitative analysis (236 posts). Forum users could have more than one post included, but only if it was a part of a unique post sequence. A single post/comment repeatedly appearing through sharing among forum participants was counted once. A thematic analysis of the posts’ content was conducted by using Framework Analysis, to organize the data to capture the patients’ perceptions, treatment effectiveness, DTE and previous therapy duration, positive effects with DTE and previous therapy, adverse effects with DTE and previous therapy, reasons for changing from the previous therapy to DTE, and related information. A step-by-step approach was taken by the research team to ensure rigor in the analysis [20]. The first step was familiarization of the data, where three researchers (FJKT, NRE, and OJP) independently reviewed and made notes on 50 posts. Next was the creation of a thematic framework where three researchers (FJKT, NRE, and OJP) read a subset of posts and developed and revised, with continuous discussions, the framework, based on emerging topics and themes arising from the posts. Following the development of the framework, an additional researcher (JPB) read a further subset of posts and approved the framework for coding. Coding was done by three researchers (FJKT, NRE, and OJP), independently and in duplicate. Similarities or differences in the coding between the three researchers were discussed and reassessed. Once coding was complete, FJKT, NRE, EHG, and OEK examined the framework within and across themes and posts, to identify the overarching themes and relationships related to our research question around patient preferences for and experiences with using DTE. All data were de-identified. NVivo 12^®^ Pro software was used to organize the posts and apply codes.

## 3. Results

### 3.1. Quantitative Analysis

#### 3.1.1. Indication and DTE Prescription Characteristics

The demographic and clinical characteristics of patients are summarized in Table 1. The most common clinical indications for DTE therapy were primary hypothyroidism/Hashimoto’s thyroiditis (51%, *n* = 257), post-surgical hypothyroidism (25%, *n* = 126), and post-ablation hypothyroidism (16%, *n* = 81). DTE doses were available in 25.6% of the posts (*n* = 172); the mean dose was 84.1 ± 56.9 mg/day, with 63% of the doses (*n* = 109) ranging between 50 and 150 mg/day. The treatment duration (available in 11.1% of the posts) was highly variable, ranging between 2 weeks to 45 years, with 54.5% of these posts describing DTE use for least than six months. Among the posts that described the source of DTE prescription, initial DTE prescription request/interest was driven mainly by the patient in 54% (*n* = 88) of the posts, followed by provider initiative (46%, *n* = 74). When the source of information of DTE was described, these posts described the main source as social networks (e.g., friends, family, and coworkers) (53%, *n* = 84), internet information sources (38%, *n* = 60), and clinicians (9%, *n* = 15).

#### 3.1.2. Previous Therapy Characteristics, Benefits, and Side Effects

Three-hundred posts (45%) described a history of prior treatment with other thyroid supplements, such as LT4 monotherapy (93%, *n* = 279), LT4 + Liothyronine (LT3) (5%, *n* = 15), or Liotrix (2%, *n* = 6). The average time on the previous therapy was 10.3 ± 8.7 years. Only 5% of the posts described any benefit with the use of the previous treatment (decreased fatigue, improvement in cold intolerance, weight loss, etc.). There were many reasons for changing from the previous medication to DTE, including no improvement in clinical symptoms (47%, *n* = 75), development of side effects (24%, *n* = 38), no change in overall well-being (22%, *n* = 36), and no changes in laboratory workup (7%, *n* = 12). The specific reasons for changing from the previous therapy to DTE in each category are shown in Table 2.

#### 3.1.3. DTE Benefits and Perceived Effectiveness

The most frequently described benefits with DTE use were an improvement in clinical symptoms (56%, *n* = 155), change in the overall well-being (34%, *n* = 94), possibility to reach previous health status (7%, *n* = 19), and a low cost compared with previous treatment (3%, *n* = 8). Among the posts describing improvement in clinical symptoms with DTE use, the symptoms associated with improvement were fatigue (28%, *n* = 43), weight gain (17%, *n* = 26), neurocognitive symptoms (5%, *n* = 8), dermatological symptoms (5%, *n* = 8) and depression (3%, *n* = 5). The average time to notice benefits with DTE treatment was 29.7 ± 32.5 days, ranging from two days to four months. Eighty-one percent of the patients considered that DTE had moderate-to-major overall effectiveness (Table 1). Moreover, the majority of the posts stated that DTE was more effective than the previous therapy (77%, *n* = 99), 13% (*n* = 17) described that DTE was as effective as the previous therapy, and 10% (*n* = 13) that DTE was less effective than the previous therapy.

#### 3.1.4. DTE Side Effects and Related Problems

Among the included posts, 136 (20%) described side effects related to the use of DTE, including weight loss (15%), fatigue (11%), palpitations (11%), heat intolerance (11%), sleep disturbances (10%), high blood pressure (7%), hair loss (5%), depression (4%), nervousness (4%), irritability (4%), tremors (3.7%), and miscellaneous (menstrual irregularities, musculoskeletal problems, etc.; 15%). The average time to notice new side effects was 64.5 ± 15.4 days. Of the posts describing the source of DTE, 63% (*n* = 75) described local pharmacies, followed by purchases outside the US (31%, *n* = 37) and online purchases (6%, *n* = 7). Some posts described logistical problems with DTE use (*n* = 77), such as availability/access difficulties (53%), the variability of the effect between batches (22%), no actual prescription (22%), and no FDA approval (2.6%).

### 3.2. Qualitative Analysis

In our qualitative exploration of a subset of forum responses, we derived five major themes: experience with previous therapies before starting DTE, perceived effectiveness and benefits of DTE, DTE side effects, the need for individualized therapy for hypothyroidism, and barriers to obtaining DTE. Table 3 provides an overview of themes and additional representative quotes for each theme.

#### 3.2.1. Experience with Previous Therapies before Starting DTE

Many participants referred to continuous fatigue and physical, and neurocognitive symptoms with LT4 treatment, despite having normal thyroid levels: “Like many others, my numbers look good, but I know how my body feels, and it wasn’t agreeing with the Synthroid”. Other participants reported that LT4 was not effective in improving their laboratory values and described a struggle to maintain normal TSH levels, despite escalating doses of LT4.

#### 3.2.2. Perceived Effectiveness and Benefits of DTE

However, after initiating DTE treatment, many participants broadly described improvement across multiple physical symptoms: “It (Armour) changed my life … I’m glad I found a medication that makes me feel normal again… all have improved; moods, skin (no itching), no head headaches, goiter is down”. Participants described DTE as a natural alternative to synthetic drugs, which may imply that it is perceived to be safe. Many participants also praised DTE as being a more economical option.

#### 3.2.3. DTE Side Effects

However, participants expressed some frustration with finding the correct dose of DTE. Some described symptoms such as insomnia, and others described abnormalities in blood tests, which were attributed to incorrect dosing. We found other posts describing a lack of efficacy or additional side effects with DTE. Some patients described symptoms such as tremors, insomnia, hair loss, and rash. Others described biochemical adverse events, such as hyperthyroidism and high glucose readings.

#### 3.2.4. Need for Individualized Therapy for Hypothyroidism

Participants also reflected their perception of DTE as a therapy that fits the patient’s context and circumstances; patients highlighted how treatment and disease characteristic change between patients. For example, one said the following: “Everyone’s battle with Hashimoto’s will be different, which will lead to different treatments. In my case, Armour has worked best”. In this context, several participants echoed the importance of patients being active self-advocates who encourage providers to consider DTE and the potential benefits of this treatment.

#### 3.2.5. Barriers to Obtaining DTE

Participants mentioned concerns related to manufacturing, such as “manufacturers stopped production”. Therefore, participants reported issues regarding the lack of pharmacy DTE dispense. These situations generate interrupted treatment and adherence. A participant said, “It’s difficult getting my prescription filled, most pharmacies don’t carry Armour anymore”. Another barrier described by the participants was provider resistance to prescribe DTE. As one participant stated, “Doctors think they know how you feel and do not even tell you about Armour. I asked my doctor and was told there were not enough studies on it to show its effectiveness”. Insurance was also described as a barrier to access to DTE, related to insurance coverage. Medicare insurance was an example mentioned due to a lack of DTE consideration as part of the insurance formulary.

## 4. Discussion

Historically, the administration of both T3 and T4, also known as combination therapy, had been long recognized as the first treatment for hypothyroidism, with many early formulations containing thyroid extracts, thyroglobulin, or desiccated animal thyroid [9,21]. Although DTE remained the cornerstone of hypothyroidism treatment for decades [22], treatment response was inconsistent and unpredictable, with reports of either continued hypothyroidism or iatrogenic thyrotoxicosis [21,23]. With the increasing use of the TSH radioimmunoassay as a treatment follow-up tool and the discovery of conversion of T4 to T3 in humans [24,25], there was a transition in clinical practice toward the adoption of LT4 as the standard of care for the treatment of hypothyroidism. Since then, LT4 monotherapy has been considered to have a favorable safety profile and greater effectiveness in normalizing the serum TSH level, the most sensitive marker of hypothyroidism-treatment response [9].

Nevertheless, there is a concern that, despite the use of LT4 at doses to reach adequate biochemical response, patients often have persistent signs and symptoms of hypothyroidism and low satisfaction with their treatment [6,8]. In this study, we observed that, among forum participants, 81% perceived as moderate-to-major the effectiveness of DTE therapy. In this regard, our results are similar to previous survey-based studies reporting higher median satisfaction; lower reports of problems with weight management, fatigue, mood, or memory [8]; and an overall positive drug effect in individuals treated with DTE, compared to those taking LT4 or LT4 + LT3 [26]. To the extent of our knowledge, there is only one double-blinded, randomized clinical trial (RCT) evaluating the effectiveness of DTE compared with LT4 in a sample of 70 patients with hypothyroidism [27]. Although this study found no differences in symptoms and neurocognitive measurements between therapies, there was a minimal, but statistically significant, weight loss after the DTE treatment period (172.9 ± 36.4 lb. vs. 175.7 ± 37.7 lb., *p* < 0.001). Moreover, upon the conclusion of the trial, 49% of the participants preferred DTE, 19% preferred LT4, and 33% had no preference. These results are in line with our findings describing a preference for DTE therapy over prior therapies and frequently described weight loss after DTE treatment.

Additionally, this trial did not show overall differences in hypothyroid symptoms improvement between DTE and LT4, suggesting that there is no clinical benefit of DTE over LT4-based therapy. In our analysis of patient forum posts, DTE use was commonly associated with improvements in overall well-being, a finding supported in the trial subgroup analyses, where those patients who preferred DTE had significant improvements in the general health questionnaire-12 and thyroid symptom questionnaire [27]. Improvements in quality of life were also previously in an observational study of 100 patients prescribed T3-containing thyroid hormone replacement (LT4 + LT3 or DTE) after receiving LT4 monotherapy, indicating reports of improved quality of life in 93% and 89% of the DTE and LT4/LT3 populations, respectively, as assessed by the Medical Outcomes Study Short Form-20 (SF-20) questionnaire [28]. In this study, more than 70% of the participants receiving DTE reported being as “healthy as anybody they knew”, 87% said “feeling calm and peaceful”, and 88% reported “being a happy person”. The results of our study are congruent with their results, as posts described an improvement in hypothyroid symptoms, change in the overall well-being, and the possibility to reach previous health status with DTE compared with prior therapy.

On the other hand, there are some criticisms with the use of DTE for the treatment of hypothyroidism, such as potential thyrotoxicosis risk and highly variable treatment response. Currently, there are two DTE preparations available in the US, and according to manufacturer’s labels, each 65 mg (1 grain) tablet of DTE contains 38 μg of T4 and 9 μg of T3 [29]. However, the ratio of T4 to T3 in these DTE preparations, once absorbed, differs from that of endogenous average human thyroid output [6,29]. Moreover, supraphysiologic fluctuations in T3 levels have been reported following DTE absorption [30]. A recent survey-based study described the adverse outcomes reported by endocrinologists and frequent prescribers of DTE products [29]. Similar to our results, among 91 reports of individuals on stable doses of DTE, adverse events were due to new or unexpected symptoms in 15 (16%), a TSH change alone in 14 (15%), or both in 62 (68%). Most of the symptoms (65%) were consistent with thyrotoxicosis, whereas five cases had symptoms typical of hypothyroidism. The occurrence of these clinical problems has been explained by an inability of titrating DTE doses reliably or by the use of DTE preparations of variable potency [29].

### 4.1. Limitations

As a forum-posts-based study, our findings have some limitations, mainly related to selection bias. We expect that the patients who experienced a markedly positive or negative effect of the DTE were overrepresented. Indeed, more than half of the posts describing DTE treatment duration showed that DTE was used for less than six months; and it is well-known that therapies are often seen positively during the initial period, which can overrepresent the positive described effects of DTE in this study. In addition, it was not possible to exclude the possibility that the same patient posted comments in different forums. Furthermore, as the diagnosis of hypothyroidism was self-reported, it is not possible to assure whether the participants of the forums do not include a significant number of individuals with misdiagnosis of hypothyroidism or are taking DTE for indications other than hypothyroidism. Many forum participants were treated for less than six months, which might increase the probability of positive perception about DTE, as often seen during the initial treatment of hypothyroidism. In the same way, there is a tendency of patients with chronic conditions, such as hypothyroidism, to associate unrelated symptoms or decreased quality of life to their thyroid condition. Due to data limitations, it was not possible to determine/include other determinants of hypothyroidism treatment response and treatment satisfaction, such as thyroid function test levels before or during DTE treatment, percentage of patients with subclinical hypothyroidism, employment status, marital status, compliance with medications, comorbidities, chronic medications, previous negative experience with health professionals, misinformation, weight, physical activity, somatic symptom disorder, etc. As we only included posts of patients taking DTE, some patients might have some beneficial effects with the use of other thyroid replacement therapies (LT4 + LT3, liotrix, etc.) that were not assessed in this study. However, this study represents an initial approach to capture first-hand patients’ experiences, perceptions, and feelings regarding DTE in a more real and unobserved setting.

### 4.2. Implications for Practice

Our study voices the patient experience of DTE treatment, allowing clinicians to appreciate and address this in treatment decisions. Interestingly, we found that about 46% of the DTE prescriptions or DTE-related patient–physician discussions were initiated by the clinician. This echoes findings of a recent survey of patients taking combined thyroid therapy, in which 44% and 41% of the respondents reported that DTE was prescribed by general practitioners or specialists, respectively [26]. Even though this conduct is controversial, DTE remains as an experimental treatment that is used at the physician’s discretion, despite the lack of recommendations in current guidelines. For example, in a simulated case scenario of a patient with low T3 and persistent hypothyroid symptoms despite adequate LT4 therapy, only about 3–6% of physicians reported that they would switch to DTE [31]. DTE consideration in this small group of physicians might represent an attitude or desire to accommodate clinical decisions to patient preferences considering patient-centered outcomes [31]. Indeed, in a previous survey of patients with hypothyroidism, individuals taking DTE reported higher median satisfaction with their current physician and perceived slightly higher levels of physician knowledge compared to patients receiving LT4 monotherapy [8]. Despite the normalization of TSH with the use of LT4, many patients continue to feel hypothyroidism-related symptoms and desire further management/treatment. Future practice guidelines should address this potential discrepancy between patients’ and clinicians’ perceptions of DTE and outline its role in practice.

### 4.3. Implications for Future Research

Although there is an indication for the preference of DTE among some patients with hypothyroidism, the reason behind this is still not well understood. Some researchers have hypothesized that high rates of patient satisfaction with DTE may be associated with underlying genetic polymorphisms (deiodinases or thyroid hormone transporters), whereby LT4 monotherapy may be inadequate but patients may respond to DTE [32,33,34,35]. These polymorphisms have been linked in retrospective studies with patients’ self-reported preference with combined thyroid therapy [35,36]. Nevertheless, no RCTs have incorporated an assessment of deiodinase or thyroid hormone transporter polymorphisms into their design when comparing the effectiveness of treatment options for hypothyroidism. Other hypotheses proposed to explain patient satisfaction with DTE include a patient preference for higher treatment doses, patients being exposed to a slightly thyrotoxic state with T3, and the presence of some other active substances other than T4 and T3 within DTE [8]. This knowledge gap highlights the need for more sophisticated and well-designed RCTs, large studies that allow for subgroup analysis to identify which patients benefit with DTE compared to LT4, and studies incorporating patient-reported and patient-important outcomes, to elucidate the physiological support responsible for combined therapy preference in this subset of patients. On the other hand, research efforts could be directed to evaluate the effect of synthetic slow-release preparations of thyroid hormones [37] in humans and the development of dose-consistent DTE preparations.

## 5. Conclusions

This study showed that patients with hypothyroidism using DTE frequently describe the lack of individualized treatments and a feeling of not been listened to as issues during their treatment. This finding reinforces the need for patient-centered approaches in current clinical practices, to consider the individual needs and the context of every patient. We also described the authentic account of a subgroup of patients who prefer DTE over LT4 for the treatment of hypothyroidism, despite its unpredictable response and potential adverse effects. Several causes might explain this finding (i.e., inadequate clinical or biochemical response with prior therapy, placebo effect, greater perceived effectiveness of DTE, and more extended benefits in overall well-being with DTE use); however, the reason behind DTE preference remains unknown and should be inquired in future investigations.

## Figures and Tables

**Table 1:** Demographic and clinical characteristics of patients.

Forums Included	*n* (%)
*Drug.com*	151 (22.4)
*PatientsLikeMe*	146 (21.7)
*Web MD*	376 (55.9)
Gender	
Female	330 (49.0)
Male	24 (3.6)
Unknown	319 (47.4)
Age (years)	
<25	8 (1.2)
25–34	26 (3.9)
35–44	56 (8.3)
45–54	102 (15.2)
55–64	97 (14.4)
65–74	55 (8.2)
>75	19 (2.8)
Unknown	310 (46.1)
DTE dosage (mg)	
<50	49 (7.3)
50–100	76 (11.3)
101–150	33 (4.9)
151–200	8 (1.2)
>200	6 (0.9)
Unknown	501 (74.4)
DTE perceived effectiveness	
Major	456 (67.8)
Moderate	88 (13.1)
Slight	45 (6.7)
None	58 (8.6)
Unknown	26 (3.9)

DTE: desiccated thyroid extract.

**Table 2:** Reasons to change from previous therapy to DTE.

	*n* (%)
**No changes in laboratory workup**	12 (7.4)
**No improvement in clinical symptoms**	75 (46.6)
Fatigue	14 (18.7)
Depression and sadness	10 (13.3)
Mental changes (concentration, memory, attention, etc.)	9 (12.0)
Hair/skin changes (hair loss, dry skin, edema, etc.)	9 (12.0)
Cold intolerance	6 (8.0)
Weight gain	6 (8.0)
Sleep disturbances	2 (2.7)
Miscellaneous (joint pain, muscle pain, constipation, anemia, etc.)	19 (25.3)
No change in overall well-being	38 (23.6)
**Development of adverse effects (according to patient perception)**	36 (22.4)
Musculoskeletal problems (joint pain, muscle pain, weakness, etc.)	5 (13.9)
Fatigue	3 (8.3)
Weight gain	3 (8.3)
Allergic reactions	3 (8.3)
Palpitations/nervousness	2 (5.6)
Gastrointestinal manifestations (nausea, vomiting, diarrhea, etc.)	2 (5.6)
Hair loss	2 (5.6)
Headache	2 (5.6)
Depression and sadness	2 (5.6)
Miscellaneous (dizziness, vertigo, hyperglycemia, etc.)	12 (33.3)

DTE: desiccated thyroid extract.

**Table 3:** Themes and representative patient quotes found in the qualitative analysis.

Theme	Representative Quote(s)
Experience with previous therapy	“Like many other, my numbers look good, but I know how my body feels, and it wasn’t agreeing with the Synthroid”. “Synthroid did not help … and gives me bad side effects … my endocrinologist blamed all side effects on everything except the Synthroid”. “Synthroid did not work … my TSH rose to 7 at low dosage then at a higher dose my TSH rose to 12”.
Experience with DTE, including perceived effectiveness and benefits	“It (Armour) changed my life … I’m glad I found a medication that makes me feel normal again … all have improved; moods, skin (no itching), no head headaches, goiter is down”. “I’ll never use synthetic medicines again”. “What I paid for one month’s supply of Synthroid is what I paid for three months supply of Armour”. “It (Armour) is less expensive than the co-pay for Synthroid”.
DTE side effects	“This was the wrong dose for me, I should have been on a much higher dose than this, and that is why it didn’t work for me this time”. “My doctor expected that this medication would help me with brain fog, energy, and tiredness. I experienced the opposite”.
Need to individualize therapy	“Everyone’s battle with Hashimoto’s will be different, which will lead to different treatments. In my case, Armour has worked best”.
Barriers to obtaining DTE	“Manufacturers stopped production [of DTE]”. “It’s difficult getting my prescription filled, most pharmacies don’t carry Armour anymore”. “Doctors think they know how u feel and do not even tell you about Armour. I asked my doctor and was told there was not enough studies on it to show its effectiveness”.
